# Microfluidics for Biotechnology: Bridging Gaps to Foster Microfluidic Applications

**DOI:** 10.3389/fbioe.2020.589074

**Published:** 2020-11-13

**Authors:** Vera Ortseifen, Martina Viefhues, Lutz Wobbe, Alexander Grünberger

**Affiliations:** ^1^Proteome and Metabolome Research, Faculty of Biology, Center for Biotechnology/CeBiTec, Bielefeld University, Bielefeld, Germany; ^2^Experimental Biophysics and Applied Nanosciences, Faculty of Physics, Bielefeld University, Bielefeld, Germany; ^3^Algae Biotechnology and Bioenergy Group, Faculty of Biology, Center for Biotechnology/CeBiTec, Bielefeld University, Bielefeld, Germany; ^4^Multiscale Bioengineering, Faculty of Technology, Bielefeld University, Bielefeld, Germany

**Keywords:** microfluidics, biotechnology, interdisciplinary research, droplet microfluidics, organ-on-a-chip, single-cell analysis, single-cell cultivation

## Abstract

Microfluidics and novel lab-on-a-chip applications have the potential to boost biotechnological research in ways that are not possible using traditional methods. Although microfluidic tools were increasingly used for different applications within biotechnology in recent years, a systematic and routine use in academic and industrial labs is still not established. For many years, absent innovative, ground-breaking and “out-of-the-box” applications have been made responsible for the missing drive to integrate microfluidic technologies into fundamental and applied biotechnological research. In this review, we highlight microfluidics’ offers and compare them to the most important demands of the biotechnologists. Furthermore, a detailed analysis in the state-of-the-art use of microfluidics within biotechnology was conducted exemplarily for four emerging biotechnological fields that can substantially benefit from the application of microfluidic systems, namely the phenotypic screening of cells, the analysis of microbial population heterogeneity, organ-on-a-chip approaches and the characterisation of synthetic co-cultures. The analysis resulted in a discussion of potential “gaps” that can be responsible for the rare integration of microfluidics into biotechnological studies. Our analysis revealed six major gaps, concerning the lack of interdisciplinary communication, mutual knowledge and motivation, methodological compatibility, technological readiness and missing commercialisation, which need to be bridged in the future. We conclude that connecting microfluidics and biotechnology is not an impossible challenge and made seven suggestions to bridge the gaps between those disciplines. This lays the foundation for routine integration of microfluidic systems into biotechnology research procedures.

## Introduction

The interest in lab-on-a-chip devices for their application in biotechnology has expanded rapidly over the past 10 years (Oliveira et al., 2016; Marques and Szita, 2017; Bjork and Joensson, 2019). Nowadays, many start-ups offer specialised microfluidic solutions for different applications and scientific questions. Over the last decade expert’s statements have been similar: “The future for microfabricated fluidics devices—or the lab-on-a-chip—looks quite promising” (Caicedo and Brady, 2016) or “Microfluidics, as an emerging technique, provides new approaches to precisely control fluidic conditions on small scales and collect data in high-throughput and quantitative manners” (Bai et al., 2018).

Nevertheless, most of the biotechnologists are still not used to integrate microfluidic systems into their typical experimental procedures in a regular manner. This challenge was already recognised 14 years ago by Helene Andersson and Albert van den Berg asking the question “Where are the biologists?” and scientists have been trying to find a solution since then. They pointed out, that technical advances in microfluidic systems have been achieved, but microfluidics researchers do still have to attract biologists’ attention. Moreover, they suggested innovative “out-of-the-box” experiments with high potential for great impact in both fields and spectacular demonstrations of new findings, which would not be achievable with conventional technologies (Andersson and van den Berg, 2006).

At the same time, the lack of a “killer application” was blamed for the missing success of microfluidic technologies within fundamental and applied research in biology (Blow, 2007; Becker, 2009; Volpatti and Yetisen, 2014). Here, a “killer application” is referred to a method that greatly outperforms current methods in regard to the desired outcome (Sackmann et al., 2014). Recently, Caicedo and Brady (2016) suggested that it is rather “bridging the gap” than looking for a killer application to bring both fields closer together, because in their opinion the “use of microfluidics is very limited beyond the academic engineering community.” They name two gaps that might explain the poor adoption of microfluidics in mainstream biomedical research and the biotech industry. This first is a lack of integration besides economic reasons and secondly the engineering of sophisticated but irrelevant microfluidic systems. As a potential solution they suggested a “thoughtful partnership” between academic engineers, biologists and industry research scientist to “increase the robustness and credibility of their findings” and that the “needs of academic life science and industrial researchers users are being met” (Caicedo and Brady, 2016).

Is that enough or are there additional approaches to be taken? Is there a general approach to bridge the gap? What are the needs of biotechnologists working in academia and industry? How can these be met by microfluidics? Moreover, looking from a practical perspective many other questions emerge.

In this review, we will take a deeper look into these questions and aim to find answers why microfluidics is still not regularly used within biotechnology labs. The goal of this article is to highlight the most relevant gaps, and thus contributing to a more balanced discussion, how microfluidics can be further integrated into biotechnology. Therefore, we briefly introduce the most important demands biotechnologists have and how microfluidics can contribute to satisfying them. We exemplarily analyse four emerging biotechnological fields that tremendously benefit from the application of microfluidic systems, namely microbial heterogeneity studies, the screening of cells, the analysis of synthetic co-cultures and organ-on-a-chip approaches. Based on the analysis, we discuss the most evident gaps and make suggestions, to enhance the integration of microfluidic systems in various research fields. The examples can serve as guideline for further discussions on how to integrate microfluidics into biotechnological procedures.

## What Microfluidics Can Offer the Biotechnologist?

**FIGURE 1:**
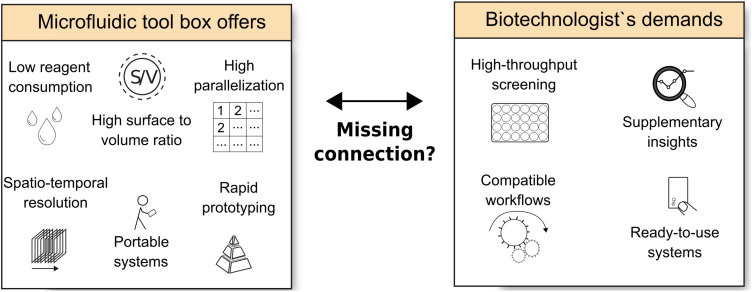
Overview of the offers of microfluidics toolboxes vs. the demands arising from biotechnological research.

To explain, why biotechnological research should profit from an inclusion of microfluidic techniques and a more intense dialogue between microfluidics and biotechnology researchers, we first list the unique strengths of microfluidics. Typically, microfluidic systems are channels filled with fluid, such as reaction media or buffers. Characteristic dimensions are the channel height and/or width in the range of few micrometres to a few hundred micrometres. The obvious advantages provided by microfluidics is the use of small volumes and precise liquid handling, which enable cost-effective high-throughput biochemical assays and diagnostics (Salieb-Beugelaar et al., 2010), but there are still others being of potential relevance for biotechnology research (Figure 1).

### Low Reagent Consumption

The first and most obvious advantage of microfluidic devices is that only very small amounts of reagents are needed due to the characteristically small dimensions. This is of high importance if expensive components, e.g., reagents or enzymes that are difficult to purify, are to be analysed. Examples are the design of new biocatalysts or antibiotics (Hage-Hülsmann et al., 2018). Additionally, the use of small volumes is advantageous in the case of hazardous reagents as their amount can be reduced in microfluidic applications (Singh et al., 2016).

### High Surface to Volume Ratio

Along with low reagent consumption comes the advantage of microfluidics that the systems provide very high surface to volume ratios. This allows fast mass and heat transport vital for various applications, e.g., biotransformation (Gervais and Jensen, 2006). For instance, microfluidics is ideally suited for applications where reactions take place at surfaces, e.g., surface immobilised enzymes or sensing applications. For sensing, often specific antibodies are immobilised to surfaces and concentrations or binding kinetics are determined by various methods like surface plasmon resonance or electric impedance (Páez-Avilés et al., 2016; Wang and Fan, 2016).

### High Spatio-Temporal Resolution

Microfluidics is a highly versatile tool for studying single cells. This is mainly due to the high spatial and temporal resolution that can be achieved. For instance, single cells or small numbers of cells can be trapped or fixed, so that the physiology of a single cell or small subpopulations can be studied over time (Greif et al., 2010; Grünberger et al., 2014). Beyond observing with high resolution, microfluidics provides the option of controlling micro-environments (Dusny and Grünberger, 2020). In this context, for example, the concentration and/or gradient of substrates can be adjusted at distinct locations due to the laminar flow (Kim et al., 2010).

### High-Throughput Applications

Due to low working volumes and the ability to parallelise reaction sites, microfluidic systems are perfectly suited for high-throughput applications such as screening experiments. A well-established and already commercially available method in microfluidics is droplet microfluidics (Teh et al., 2008). In these devices, water-in-oil or oil-in-water droplets are generated at a controlled volume and speed. Small compartments provide the ability to screen cells or new enzymes in a high-throughput manner, comparable to FACS (fluorescent activated cell sorting) (Kaushik et al., 2018; Wang et al., 2019). The advantage of droplet-based enzyme screening is that the enzymes are encapsulated in a small environment, and thus even small product amounts are detectable, due to high local concentrations (Colin et al., 2015; Bornscheuer, 2016). This also applies to whole-cell biocatalysts, which immediately excrete the products. In contrast to conventional methods like FACS, microfluidic devices provide excellent abilities for high parallelisation due to their small dimensions. Thereby, a high number of parallel experiments can provide a sample size allowing drawing statistically sound conclusions from a single run.

### Rapid Prototyping

Microfluidic devices used in academic research are most often custom-made based on PDMS [poly(dimethylsiloxane)] soft lithography. This technique allows rapid prototyping and thus flexible adaptation of the device to the specific needs of individual experiments (Sia and Whitesides, 2003). In recent years, new fabrication techniques were established for fast prototyping with different types of co-polymers; e.g., the milling of microstructures (Guckenberger et al., 2015), or 3D-printing systems (Viefhues et al., 2017; Weisgrab et al., 2019).

### Portable Systems

When microfluidics was invented, the first aim was to develop a system that incorporates all instrumentation and analysis features of a laboratory but in a hand-held format to provide a portable system. Though this research field has developed over the past 30 years immensely, this aim is not fully realised yet. Nevertheless, the small dimensions of the devices allow, to some extent, experiments at varying locations of interest, e.g., for point of care and point of use testing (Wang et al., 2017, 2019).

**FIGURE 2:**
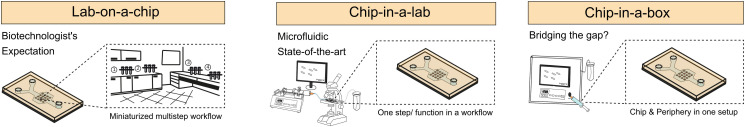
The discrepancy between biotechnologists’ expectations and the state-of-the-art in microfluidics. One suggestion for bridging the gap and the missing connection between both fields could be a “chip in a box” solution, combining microfluidic chip and all necessary periphery in one setup for conducting the experiment.

Despite the various advantages microfluidics offers, there is a clear discrepancy between the experimenter‘s expectations and the actual capacity of available devices (Figure 2). Biotechnologists, using microfluidics, and even microfluidics have always been dreaming of a single lab-on-a-chip device enabling the measurement of multiple parameters or the automatised processing of a multi-step workflow (Figure 2). However, even state-of-the-art microfluidic devices do not provide multiple functions and are often tailor-made systems that operate on a proof-of-concept status. Most of these microfluidic systems can be seen as a “Chip-in-a-lab” solution (Figure 2; Streets and Huang, 2013; Mohammed et al., 2015), performing selected steps within already existing workflows. To be functional, different ancillary devices (e.g., pumps, microscopes) are necessary to perform the desired task.

## Which of the Current Demands in Biotechnology Can Be Addressed by Microfluidics?

Biotechnology is a highly diverse field in which enzymes, cell extracts or whole organisms are used for technical applications and the production of valuable compounds (Thieman and Palladino, 2019). While white biotechnology is devoted to produce industrially relevant products in a cost- and time-effective manner, red biotechnology is very much focussed on medical applications, e.g., therapeutic proteins or organ-on-a-chip devices. Blue biotechnology exploits marine bioresources, while green biotechnology uses photosynthesising microalgae and plants to convert inorganic carbon into various products. Other, more recent fields of biotechnology like grey (environmental) and yellow (insect) biotechnology should also be mentioned, since theses disciplines also rely on cultivated cells. Considered that biotechnology uses different types of organisms, the ways of implementing microfluidics into research projects naturally also differs. In the following section, we try to identify which kind of microfluidics, relevant for any type of research with single-celled organisms (heterotrophic bacteria, mammalian cells or phototrophic microbes), a biotechnologist demands (Figure 1).

### Addressing the Demand for Increased Screening Throughput

In white biotechnology it is of utmost importance that production strains have a high capacity to produce compounds of interest, which result from iterative strain engineering approaches, consisting of repeated mutagenesis and selection cycles. Especially, in situations where mutants are created by random integration of transgenes into nuclear genomes, mutant libraries in the range of several hundred (Wichmann et al., 2018) to several thousand (De Jaeger et al., 2014) mutants have to be analysed in order to identify desired phenotypes.

In other biotechnological areas the demand for powerful screening methods is even greater. The directed evolution of enzymes helps creating bespoke enzymatic activities to improve their suitability for industrial processes. Techniques such as random mutagenesis or gene shuffling are applied (Arnold, 2019) to create mutant libraries which easily reach the complexity of >10^12^ variants (Galán et al., 2016). Frequently, several simultaneous mutations have to be present in order to observe the desired catalytic effect (Markel et al., 2020). This diversity calls for novel high-throughput screening methods, because conventional techniques like microtitre plates only allow the analysis of up to 10^4^ variants per day (Xiao et al., 2015), while agar plate-based assays can process library sizes of up to 10^5^ (Leemhuis et al., 2009; Tee and Wong, 2013). Similarly, microbial consortia can be a treasure trove for novel compounds or enzymes of high biocatalytic potential but need to be screened with sufficient tools guaranteeing sufficient throughput (Lee et al., 2019).

### Addressing the Demand for Supplementary Insights Beyond the Bulk

In standard experiments, i.e., without using microfluidics, biotechnologists traditionally investigate the response of cells of a given organism by analysing a whole cell population, thus looking actually at an “averaged response,” masking the indisputable phenotypic and genotypic heterogeneity present in shake flasks or bioreactors (Lidstrom and Konopka, 2010). As an example, cell-to-cell heterogeneities can be detrimental for the stability and the overall performance of production processes and understanding them at the molecular level should help avoiding these phenomena (Xiao et al., 2016). Existing methods such as flow cytometry reveal insights into population heterogeneity but additional insights into dynamic single cell-behaviour are not provided (Dusny and Grünberger, 2020).

Novel tools that provide insights into dynamic processes of cells with full temporal resolution would thus be beneficial. Therefore, it is of central interest to establish novel approaches with single-cell resolution, which are currently performed in bulk measuring “averaged response.” This includes single-cell omics technologies such as single-cell sequencing, single-cell transcriptomics (Rich-Griffin et al., 2020) or single-cell proteomics (Wang and Bodovitz, 2010; Lazar et al., 2019; Marx, 2019).

The cultivation of cells is traditionally performed in bulk scale. Although the cultivation conditions can be defined as “well-controlled,” micro-gradients within different environmental parameters exist (Delvigne and Goffin, 2014). This includes gradients in nutrient concentration (Demling et al., 2018), CO_2_ pressure (Mostafa and Gu, 2003) or light gradients (Jacobi et al., 2012). These effects even increase during scale-up of the cultivation scale (Crater and Lievense, 2018).

Thus, novel methods that enable the cultivation of cells under defined and/or constant environments are of central interest. First, this will enable the investigation of cellular physiology in a precise manner. Second, such methods could be used to mimic complex environmental conditions such as those found in nature or technical cultivation systems (Täuber et al., 2020).

### Addressing the Demand for Compatible Ready-to-Use Microfluidic Analysis Devices

Work of biotechnologists and of other experimental researchers is often limited by the accessibility of equipment, which imposes restrictions to their experimental design. New technologies such as microfluidics should provide solutions for handling lab routines within a single device (lab-on-a-chip), which is affordable and can analyse various parameters per experimental run. In addition, the microfluidic system should allow integration into the existing experimental procedure (Dusny and Grünberger, 2020).

Most biotechnologists seek for solutions, which provide the whole analytical workflow in one step. This demand was already recognised and simplified, versatile devices are increasingly designed to satisfy the needs of the end-users. The establishment of standard unit operations and the possibilities to carry out experiments in a biological context give the opportunity to design more complex workflows to address biological research challenges (Kintses et al., 2010). Ideally, “chip-in-box” systems are available, where microfluidic platforms, control infrastructure and analysis technology are implemented into one bench-top device (Streets and Huang, 2013). Thus, experimentalists do neither need additional periphery such as pumps, nor control or analysis units. Avoiding the need for costly equipment offers a chance for microfluidics to find its way into smaller laboratories.

Researchers including biotechnologists are more and more forced to obtain experimental results quickly. The availability of ready-to-use systems is thus beneficial. Therefore, there is a high demand for improvements of microfluidic devices, which often possess a proof of concept status.

## Case Studies to Analyse Missing Connections of Microfluidics and Biotechnology

Four distinct fields were selected to illustrate how microfluidic systems found application in biotechnology. As an established field, cell screening was selected, based upon its scientific relevance, while three emerging fields (heterogeneity, organ-on-a-chip and mixed cultures) were chosen due to their assumed innovation potential. Since the 2000’s the field of single cell analysis is continuously growing, which resulted in more than 2000 publications appearing in topic-specific database search using Web of Science (Clarivate Analytics) with the keyword combination “single cell” plus “microfluidics.” Single-cell analysis can be split into many different subgroups (Gao et al., 2019). The most important subgroup is the application-oriented topic of cell screening, which started in the 2000’s based on the technological development of droplet microfluidics (Teh et al., 2008). In total, 1286 publications were found combining the keywords screening and microfluidics since 2000, of which 43% were published within the last 3 years.

Since 2010, heterogeneity studies of cell populations (Schmid et al., 2010) and organ-on-a-chip applications (Marx et al., 2012) are emerging and represent two growing topics within the research community. Lately, the interest in using microfluidic single-cell systems for the investigation of mixed cultures is growing (Burmeister et al., 2018; Burmeister and Grünberger, 2020).

### Achieving Ultrahigh-Throughput Cell Screening Capacity With Microfluidic Devices

Motivated by the new advances within microfluidics, allowing scientists to analyse communities on a single cell level, screening approaches seeking for high producers came into the focus of biotechnologists. Up to now, most screening approaches on single cell levels were carried out using traditional single cell fluorescence activated cell sorting (Becker et al., 2008). The big and inherent disadvantage of this approach is the exclusive detection of signals within the cell (Wang et al., 2014). Therefore, the method is not suitable for products that are secreted. However, considering costs and ease of downstream processing, product excretion is frequently the preferred strategy. An alternative to FACS is droplet microfluidics using water/oil/water emulsions. Recently, a study compared both methods with regard to an improved production phenotype for riboflavin in *Yarrowia lipolytica*. The adaptive evolution study demonstrated that screening via single cell FACS favoured the selection of strains with high intracellular riboflavin accumulation, while droplet FACS primarily led to the identification of strains with a high riboflavin secretion capacity. Based on these results, the authors concluded that microdroplet-enabled FACS possesses great potential for strain engineering (Wagner et al., 2018).

In recent years, studies using high- and ultrahigh-throughput screening in droplet-based microfluidics targeted directed enzyme evolution (Agresti et al., 2010; Zeymer and Hilvert, 2018), selection for specific phenotypes (Wang et al., 2014; Beneyton et al., 2016) and desired products (El Debs et al., 2012; Sjostrom et al., 2014; Wagner et al., 2018).

The directed evolution of enzymes in order to tailor substrate specificity, regio- and enantioselectivity or robustness (e.g., thermotolerance) does frequently also rely on the screening of cells. Monodisperse water-oil (w/o) droplets are a system that can be used to entrap single cells in a compartment which contains all reagents necessary for the screening reaction (substrates, buffers, cell lysis reagents, fluorescent dyes, etc.) (Markel et al., 2020). Monodisperse w/o droplets have been successfully used in microfluidic chip environments to sort droplets based on enhanced enzyme activity prior to direct DNA recovery (Kintses et al., 2012), droplet generation and sorting on a single chip (Obexer et al., 2017) or sorting, which requires two distinct substrates and fluorescent signals (Ma et al., 2018).

Those studies exemplarily show how microfluidics, especially droplet-based microfluidics already meet the demands of biotechnologist’s for high-throughput screening. Nevertheless, there is no routine use of microfluidic devices in screening processes.

But what limits the application of these microfluidics-based screening techniques?

Although, it can be envisioned that droplet microfluidics will become an indispensable tool in biotechnology for screening large cell libraries (Suea-Ngam et al., 2019), technological advances are necessary to bridge the gap from micro-scale screening to biotechnologically relevant scales. Moreover, the integration into work routines and the broad acceptance in the biotechnology community can be facilitated by establishing the necessary competences to handle these platforms and gradually improve functionality and the distribution of microfluidic knowledge. A possible short-cut for fasten the integration into lab-routines would be the yet missing commercially available plug and play solutions (Hengoju et al., 2020).

### Heterogeneity—Exclusive Insights Into Population Dynamics

Microfluidic single-cell cultivations harbour a tremendous potential for research on population heterogeneity (Dusny and Schmid, 2015). Artificial microbial habitats, cultivation modes, methods in data acquisition and analysis can be applied in a modular manner offering outstanding insights into population dynamics, usually overseen within bulk measurements (Lidstrom and Konopka, 2010). Monolayer growth chambers and so-called mother machines (Mather et al., 2010; Grünberger et al., 2015) are regularly applied to understand diverse cellular processes at the single-cell level, ranging from growth (Wang et al., 2010), stochastic gene expression (Kaiser et al., 2018), ageing (Lee et al., 2012), metabolic cross-feeding (Moffitt et al., 2012; Burmeister et al., 2018) to quorum sensing (Prindle et al., 2011).

Novel insights already change the view onto metabolic processes such as diauxic shifts (Boulineau et al., 2013; Solopova et al., 2014) or metabolite production (Mustafi et al., 2014). During the lag-phase within bulk cultivations, the majority of cells show growth arrest upon switches of carbon sources. Boulineau et al. (2013) could show, that a significant fraction of cells (∼15%) maintained high elongation rates without any detectable lag phase, which was due to the fact that these cells were already expressing the *lac* gene as a result of stochastic processes. Mustafi et al. (2014) showed a significant heterogeneity within growth and production during L-valine production of *C. glutamicum* (Mustafi et al., 2014). These insights have been masked during conventional analysis. Both studies exemplarily show how microfluidic single-cell tools can contribute to an improved understanding of microbial heterogeneity, which would not have been possible with conventional technologies.

Despite these examples of successful microfluidics application, a routine use has not been established. This cannot be satisfyingly explained by the technique not being ready for a more widespread use. Scientists have learned to apply the soft lithography technology for fabrication of disposable PDMS chips. Students can learn the basic technology quite simple, since the methodology and technology has advanced to a ready-to-use technology for rapid prototyping of microfluidics chips (Xia and Whitesides, 1998). We deem the main factor which prevents a systematic use to be a lack “motivation,” since bulk measurements are accepted as valid and are common within the biotechnology community. From a technological perspective, only a missing automation and image analysis pipelines limit its routine use of microfluidics. More user-friendly systems and automated analysis workflows will likely increase the frequency of application.

### Organ-on-a-Chip—Avoiding Animal Testing

Drug development needs to tackle several hurdles before a new drug is certified, e.g., by the U.S. Food and Drug Administration (FDA). During the approval process numerous preclinical tests have to be performed to evaluate the desired besides unwanted side-effects. So far, animal testing is the established method for those tests with the known disadvantages, e.g., transferability of the results. Thus, the need for alternative preclinical tests is tremendous. So called organ-on-a-chip (OOC) devices enable studying the effects of pharmaceutical agents and the development of disease models for the organ of particular relevance (Prantil-Baun et al., 2018). Several groups reviewed the organ-on-a-chip topic, highlighting the advantages of the techniques and the current challenges (Huh et al., 2011; van der Meer and van den Berg, 2012; Wikswo et al., 2013; Zhang and Radisic, 2017).

The microfluidic OOC devices consist of (small compartments of) human organ cells and characteristic surroundings. For instance, a lung-on-a-chip consists of structures that provide periodic stretching of the cells, a membrane support that separates two cell species, i.e., endothelial and epithelial cells, ventilation and fluid perfusion (Huh et al., 2010). Maschmeyer et al. (2015) demonstrated a microfluidic system that provided long-term co-culture of four human organs, i.e., intestine, liver, skin, and kidney. Such a system could be used for testing the impact of new drugs on the respective organs, thus being of very high importance for future medical and pharmaceutical research. Studies with OOC devices provide new insight into (complex) cell interactions of different cell types, like endothelial and neural cells (Maoz et al., 2018). The first multi-organ devices have been demonstrated successfully. The first commercial OOC devices are available and in use (Hübner et al., 2018; Sances et al., 2018; Kane et al., 2019).

Organ-on-a-chip systems are an example for the successful interdisciplinary cooperation between microfluidics and medical researchers. Communication and the associated transfer of knowledge between the disciplines works very well. This is triggered by the demand for new technologies in drug testing, since old methods/technologies are subject to further restrictions, like the Animal Welfare Act revised in 2008. To date, only very few Organ-on-a-Chip systems meet end-user usability requirements (Junaid et al., 2017). It can be assumed that a further development of the technology affecting more sophisticated applications, such as integration of on-chip sensing and the analysis of excreted metabolites will result in a plenitude of ready-to-use systems available in the commercial market (Rothbauer and Ertl, 2020).

### Mixed Cultures—Mimicking Microbial Communities and Environments

Outside the controlled, artificial lab environment, microorganisms thrive within generally quite complex multi-species communities (Boetius et al., 2000). This is of great biotechnological relevance since (engineered) communities can fulfil synthesis and degradative tasks not realisable by an individual species alone (Hays et al., 2015). Furthermore, the existence of a “microbial dark matter” containing highly interesting species, whose existence is predicted by metagenome analyses, but which cannot be cultivated using current cultivation techniques (Bernard et al., 2018), calls for a deepened understanding of interspecies interactions. Studies conducted within the last decade clearly demonstrated that microfluidic co-cultivation systems can significantly contribute to the improved understanding of factors shaping microbial communities (Nichols et al., 2010; Nagy et al., 2014, 2018).

Microfluidic systems can provide a defined microenvironment, single-cell resolution and offer either contact-based or contactless studies. Microwells, providing a defined spatial structure and enabling chemical communication between consortial members stabilised a syntrophic minimal community (Kim et al., 2008). Microfabricated habitats were used to analyse spatial impacts on the dynamics of a two-component community comprising a “cheater” and a cooperating bacterium. In their study, Hol et al. (2013) showed that provision of a spatially structured habitat prevents the dominance of the cheater, paving the way to explanations, why in natural communities such members can be kept in check. Various coupled microchambers, which physically separate cells but still allow chemical coupling, were applied to investigate responses of chemotaxis or metabolite exchange in bacterial populations (Moffitt et al., 2012; Nagy et al., 2014; Burmeister et al., 2018).

The rather young research field working on the connection between microfluidics and mixed cultures with few groups yielding in 73 articles and 6 reviews (Web of Science) has a high potential. So why does only a small fraction of groups work with “mixed cultures” use microfluidics more frequently?

Although many factors simultaneously control community composition (Hays et al., 2015), the existing microfluidic technology can rather present a small part of microbial systems than mimicking complex environments. This leads to a methodological gap, since the important question arises, if results obtained in small-scale microfluidic environments sufficiently mimic the natural habitat.

Overall, these examples indicate already the enormous future potential of microfluidics platforms as an experimental environment to study metabolite exchange, physical interaction, landscape colonisation and the impact of the microenvironment on co-culture (synthetic community) stability.

## Discussion and Conclusion

### Defining and Bridging the Gaps Between Biotechnologists and Microfluidics

**FIGURE 3:**
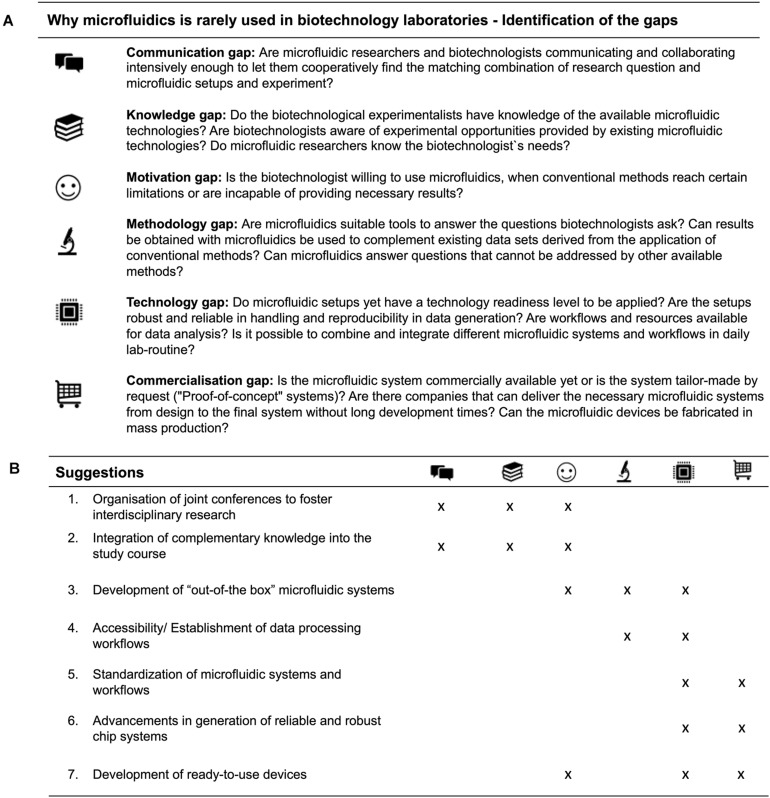
Identification of respective gaps, that prevent the interdisciplinary research **(A)** and suggestions to bridge the discussed gaps between microfluidics and biotechnology in the future **(B)**.

Biotechnological interest in the application of microfluidics has expanded rapidly and numerous studies show the potential of microfluidic systems for biotechnological research. We discussed the inherent advantages of microfluidic systems (Figure 1) for their use in biotechnology and worked out the case-specific relevance in specific research fields. Based on the presented microfluidic offers, the demands of the biotechnologist‘s (Figure 1) and the discussed case studies, several central gaps can be determined and defined that might explain the poor integration of microfluidics into biotechnological research (Figure 3A): communication gap, knowledge gap, motivation gap, methodology gap, technology gap and commercialisation gap.

Those central gaps need to be addressed and discussed in order to push forward the application of microfluidics in biotechnological research projects. But how can the defined gaps be bridged that prevent both fields from merging? (Figure 3B).

In order to bring together the current generation of scientists, joint conferences of biotechnologist’s and researchers within the field of microfluidics should be organised to foster interdisciplinary research. A lively exchange, broad discussions and information about current developments not only help to overcome the communication barrier, but also to increase knowledge about the other research area. A second suggestion to foster the communication as well as the knowledge between microfluidic engineers and biotechnologist‘s in the future is the integration of microfluidic courses into the biotechnological degree programmes and *vice versa*. Students developing microfluidic devices should be guided to meet the expectations and demands of biotechnologists. Young researchers studying biotechnology should be aware of the possibilities microfluidic tools offer as well as of the challenges in the development of this rather new technology. Alternatively, one could consider the implementation of completely new study courses such as “Biomicrofluidics,” “Bioprocessmicrofluidics,” or “Microfluidics for life sciences,” which cover fundamentals and details of both disciplines. The interdisciplinary study course “Biomechatronics” can serve as a blueprint for operation. This lays the foundation for fruitful collaborations of early-stage researchers in the future.

Our third suggestion addresses the point, that microfluidic researchers should consider the biotechnologists’ demand and develop unconventional and creative solutions (“out-of-the-box” microfluidic devices). It is of upmost importance, that novel microfluidic methods exceed the functionality of already established methods. Alternatively, there should be a clear advancement in throughput or price. One other main driver in the integration and application of new technologies is if those are saving experimental time.

Our fourth suggestion addresses the need for accessible and established data processing workflows. Currently, analysis workflows for most tailor-made microfluidic systems do not exist. To push a superfluous space methods further forward towards end-user application, not only microfluidic setups but also analysis workflows need to be established and accessible in an user-friendly manner. Unfortunately, existing tools and workflows are often difficult to transfer and adapt to new applications. For example, tools for the analysis of image data obtained by microfluidic single-cell cultivation and live cell imaging are available (Leygeber et al., 2019), but need to be adapted for every specific application. Even when a suitable tool or workflow has been found, there is often a lack of a generally accessible infrastructure for processing large amounts of data. While devices are becoming faster and better and thus generating more and more data, their processing is also becoming more complex and the necessary skills are often lacking. Advancements in technology development for example bioinformatics and novel machine learning approaches for data analysis will enable handling of large data sets and thus accelerate the analysis and application of microfluidic systems in biotechnology.

Another approach to overcome the mentioned gaps will be the standardisation of microfluidic systems and workflows. Currently a vast amount of microfluidic systems and workflows exists, and microfluidic consumable providers are only able to address part of the overall analysis system. End-users often have to setup platforms based on different suppliers, which is often incompatible. This makes operation, but also comparison of experimental results quite difficult. Therefore “World-to-chip” interfaces and basic microfluidic operations should be standardised. Here, the development of standardised microfluidic modules could be helpful to bridge the gap for reproducible, easy to operate, and building block systems (Dekker et al., 2018). Moreover, companies should offer a large portfolio of standardised systems and workflows for end-user integration.

Another important point is the development of reliable and robust chip systems/solutions (suggestion 6). In academia current microfluidic systems are highly specialised and often lack adaptability and reliability. Most microfluidic chips require a dedicated, often complex periphery, consisting of chip holders, tubing, pumps, and high-end readout interfaces (see Chip-in-a-lab). Consequently, these devices must be operated by trained technicians (in many cases their inventors), who have the necessary skills and time to set up, monitor, and continuously troubleshoot running systems, sometimes over the course of night-long experimental sessions. The investment of money and time in establishing and integrating a new technology is accompanied by the expectation of a guaranteed 24 h operational robustness and reproducibility. These expectations must be met in order to avoid slowing down the introduction of microfluidics in a large number of biotechnological laboratories.

Finally, we suggest putting emphasise on the development of user-friendly and ready- to-use lab-on-a-chip devices which are compatible with biotechnological procedures. We predict, that commercially available and ready-to-use microfluidic devices will increase the motivation of the users to adopt new techniques in their daily lab routine. This only can be addressed if microfluidic companies and microfluidic researchers closely cooperate during development of (new) microfluidic devices. Along with research and development it should be considered that separate devices serve as building-blocks (standardisation) that are compatible with each other and are at the same time ready-to-use in a plug-and-play manner. Furthermore, the chip design and its material should be capable for mass fabrication. For instance, a change from the typical PDMS chips, frequently used in academics, to e.g., polystyrene injection moulding or emerging 3D printing has to be considered. There is a clear demand for purchasable systems, facilitating a wide-spread implementation of microfluidics in this emerging discipline of biotechnology and the integration in daily lab routines. Moreover, the acceptance of new technologies is increased if they provide user-friendly software including a clear user interface and technical support. Biotechnologists on the other hand, need to be aware of the efforts and time necessary to develop such devices, though. This can prevent misunderstandings and also disappointments. The ideal realisation would be chip-in-a box solutions which would offer easy handling, ready-to-use experimental workflows that can be easily operated by biotechnologist that are unexperienced by performing microfluidic experiments.

## Conclusion

Bridging the gap to enhance interdisciplinary research between microfluidics and biotechnology is not an impossible challenge. We conclude that there are already a large number of successful collaborations linking the two disciplines. The technical advancements taking place in microfluidics, as well as biotechnological applications clearly showcase the potential for ground-breaking research. Interestingly, as more research groups and companies adopt microfluidic approaches, more creative solutions and applications arise. Given that gaps are bridged by the above made suggestions, microfluidics has a tremendous potential, providing powerful platforms for biotechnological research. However, it is still hard to predict when microfluidics will be a technique fully established in almost every biotechnologist’s lab in near future.

## Author Contributions

VO, LW, MV and AG contributed to conception and design of the study. VO and LW designed and wrote the biotechnological manuscript sections. MV and AG designed and wrote the microfluidic sections of the manuscript. VO designed all Figures of the manuscript. All authors contributed to manuscript revision, read, and approved the submitted version.

## Conflict of Interest

The authors declare that the research was conducted in the absence of any commercial or financial relationships that could be construed as a potential conflict of interest.
